# Disparities in COVID-19 testing and outcomes among Asian American and Pacific Islanders: an observational study in a large health care system

**DOI:** 10.1186/s12889-023-15089-w

**Published:** 2023-02-06

**Authors:** Jiang Li, Davis Chhoa, Latha Palaniappan, Kevin J. Hays, Alice Pressman, Nancy E. Wang

**Affiliations:** 1 Palo Alto Medical Foundation Research Institute, Center for Health Systems Research, Sutter Health, 795 El Camino Real, Palo Alto, CA 94301 USA; 2 Stanford University School of Medicine, Stanford, USA; 3 Stanford University School of Medicine, Primary Care and Population Health, 900 Blake Wilbur Dr Rm W200, 2Nd Fl MC 5358, Stanford, CA 94304 USA; 4 Departments of Emergency Medicine and Pediatrics, Stanford University School of Medicine, 900 Welch Road - #350/MC: 5768, Palo Alto, CA 94304 USA

**Keywords:** COVID-19, COVID-19 Testing, Asian Americans, Ethnic and racial minorities, Morbidity, Hospitalization, Health services accessibility, Immigrants

## Abstract

**Background:**

The COVID-19 pandemic has disproportionately impacted racial and ethnic minorities in the United States, including Asian Americans, Native Hawaiians and Pacific Islanders (Asian Americans and NH/PIs). However, few studies have highlighted nor disaggregated these disparities by Asian Americans and NH/PIs ethnic subgroups.

**Methods:**

This retrospective, cross-sectional observational study aimed to assess variation of Asian Americans and NH/PIs COVID-19 testing and outcomes compared to non-Hispanic Whites (NHW). The study utilized data from the electronic health records (EHR) and the COVID-19 Universal Registry for Vital Evaluations (CURVE) from all patients tested for SARS-CoV-2 (*n* = 556,690) at a large, health system in Northern and Central California between February 20, 2020 and March 31, 2021. Chi-square tests were used for testing differences in the severity of COVID-19 (hospitalization, ICU admission, death) and patient demographic and clinical characteristics across the Asian Americans and NH/PIs subgroups and NHW. Unadjusted and adjusted Odds Ratios (ORs) were estimated for measuring effect of race ethnicity on severity of COVID-19 using multivariable logistic regression.

**Results:**

Of the entire tested population, 70,564/556,690 (12.7%) tested positive for SARS-CoV-2. SARS-CoV-2 positivity of Asian subgroups varied from 4% in the Chinese and Korean populations, to 11.2%, 13.5%, and 12.5% for Asian Indian, Filipino, and “other Asian” populations respectively. Pacific Islanders had the greatest subgroup test positivity at 20.1%. Among Asian Americans and NH/PIs patients with COVID-19 disease, Vietnamese (OR = 2.06, 95% CI = 1.30–3.25), “Other Asian” (OR = 2.13, 95% CI = 1.79–2.54), Filipino (OR = 1.78, 95% CI = 1.34–2.23), Japanese (OR = 1.78, 95% CI = 1.10–2.88), and Chinese (OR = 1.73, 95% CI = 1.34–2.23) subgroups had almost double the odds of hospitalization compared to NHW. Pacific Islander (OR = 1.58, 95% CI = 1.19–2.10) and mixed race subgroups (OR = 1.55, 95% CI = 1.10–2.20) had more than one and a half times odds of hospitalization compared to NHW. Adjusted odds of ICU admission or death among hospitalized patients by different Asian subgroups varied but were not statistically significant.

**Conclusions:**

Variation of COVID-19 testing and hospitalization by Asian subgroups was striking in our study. A focus on the Asian Americans and NH/PIs population with disaggregation of subgroups is crucial to understand nuances of health access, utilization, and outcomes among subgroups to create health equity for these underrepresented populations.

**Supplementary Information:**

The online version contains supplementary material available at 10.1186/s12889-023-15089-w.

## Background

Racial and ethnic minorities are disproportionately affected by all aspects of the COVID-19 pandemic. While racial ethnic disparities in African American, Native American, and Latino communities have been highlighted, relatively few studies have been published regarding Asian Americans, Native Hawaiians and Pacific Islanders (Asian Americans and NH/PIs Asian Americans and NH/PIs) living in the United States (US), and even less has been published regarding diverse Asian Americans and NH/PIs subgroups [1, 2]. Some studies have noted disproportionate rates of serious outcomes including mortality in Asian Americans and NH/PIs as a whole; however these findings have not been consistent [3–6]. Understanding the effects of the pandemic on the 22 million Asian Americans and NH/PIs living in the US is particularly limited because this highly racially and ethnically heterogeneous population is generally depicted under the single category of “Asian American and Pacific Islander” [7].

The Asian Americans and NH/PIs population consists of more than 50 Asian and Pacific Islander ethnic groups who speak over 100 languages. Within the Asian Americans and NH/PIs population, there are significant differences in language, religion, culture, immigration history, socioeconomics, demographics, and health profiles that are reflected in the unique experiences of each ethnic subgroup [8, 9]. Different Asian Americans and NH/PIs immigrant populations came to the US in different time periods, for different purposes and settled in different areas [7, 10]. Asian subgroups have experienced different types of structural racism, trauma and health effects from these histories [11]. Immigration histories can profoundly affect health experiences and outcomes. Hawaiian and Pacific Islanders experience health disparities that are different than that of Asian Americans, but these are masked when lumped together as Asian Americans and NH/PIs. Native Hawaiian and Pacific Islanders have increased rates of smoking, alcohol consumption, and obesity; are more likely to be diagnosed with heart disease than non-Hispanic Whites (NHW) and Asian Americans; and are more likely to be diagnosed with diabetes than NHW [12, 13]. When compared to NHW, Filipino Americans are three times more likely to have hypertension, two times more likely to have diabetes, and three times more likely to be overweight or obese [14]. Relatively increased incomes and educational levels of Chinese and Japanese aggregated into Asian Americans and NH/PIs data support a “model minority myth;” decreased incidence of some morbidities perpetuate the assumption of a “healthy immigrant effect” and obscure health statistics unique for different Asian subgroups [15].

To date, understanding of COVID-19 disease outcomes within the Asian Americans and NH/PIs population has been contradictory and lackluster. Despite Asian Americans and NH/PIs being the fastest growing racial/ethnic group in the US, with the largest subgroups being Chinese, Asian Indian, and Filipino, change in standards of the classification of race/ethnicity [16], and knowledge about the differences in experience [8, 9], the lack of Asian Americans and NH/PIs data and minimal disaggregation of available data has limited our knowledge of the impacts of COVID-19 on Asian Americans and NH/PIs [17, 18]. During the beginning of the pandemic, COVID-19 reports and studies excluded data on Asian Americans, while other studies highlighted the disproportionate burden of COVID-19 disease on Asian Americans [19, 20]. We postulate that different findings from Asian Americans and NH/PIs populations are due to a diverse population with different subgroup effects obscuring each other [21].

We utilized data from a large healthcare delivery system in Northern and Central California to assess the variation of the COVID-19 testing and outcomes among the six largest Asian subgroups (Asian Indian, Chinese, Filipino, Japanese, Korean, Vietnamese) and “other Asians” (e.g., Cambodian, Hmong, Laotian, Pakistani, Tai) Pacific Islanders (Native Hawaiian, Guamanian/Chamorro, Samoan and other Pacific Islanders such as Fijian, Tongan), and “mixed Asian Pacific Islanders” compared to non-Hispanic Whites (NHW) (9 Asian Americans and NH/PIs subgroups). We hypothesized that subgroups of Asian Americans and NH/PIs patients with COVID-19 would be more likely than NHW patients to have serious outcomes including hospitalization, intensive care unit (ICU) admission, or death.

## Methods

### Study setting

This study was conducted at Sutter Health, a community-based health system covering 22 California urban and rural counties, including the 10 counties in the San Francisco Bay area. Sutter Health delivers comprehensive medical services in more than 100 ambulatory clinics and 24 acute care hospitals and serves an estimated 3.5 million people each year. As of 2020 Sutter patients self-identified their race/ethnicity in the electronic health record (EHR) as: 45.6% White, 15.6% Hispanic, 16.5% Asian, 4.7% Black/African American and 17.4% other [22].

### Data sources

We utilized two data sources: 1) The Sutter Health Epic EHR (Epic Systems Corporation), which is integrated across all hospitals and ambulatory sites and contains socio-demographic as well as real-time clinical data. 2) The Sutter Health COVID-19 Universal Registry for Vital Evaluations (CURVE), a semi-real-time registry of all confirmed and suspected cases of COVID-19 at Sutter Health.

### Study sample

For this retrospective cross-sectional study, we used data for patients who had a reverse transcription polymerase chain reaction (RT-PCR) test for SARS-CoV-2 performed at a Sutter Health facility between February 20, 2020 and March 31, 2021. We first identified all patients tested (n = 556,690), then extracted all Asian Americans and NH/PIs (76,892//556,690 = 13.8%) and NHW patients (comparison group, 276,088/556,690 = 49.6%). COVID-19 cases were identified from CURVE and defined as having one or more positive tests for SARS-CoV-2. These patients were then linked to patient encounter-level data extracted from the EPIC Systems electronic health records 12 months prior and up to 15 months afterwards to initial diagnosis. The patient identifiers have been removed from these datasets before analysis.

### Measures

For all Asian Americans and NH/PIs and NHW COVID-19 cases, we extracted demographic information including age, sex, race/ethnicity, primary insurance and address. Age was classified as < 18, 18–39, 40–59, 60–79 and 80 + years. We subdivided the Asian Americans and NH/PIs population into Asian subgroups (Asian Indian, Chinese, Filipino, Japanese, Korean, Vietnamese), other Asian, Pacific Islanders, and mixed Asian Pacific Islanders. Patient primary insurance was classified as commercial (HMO or PPO/FFS), public-Medicaid, public-Medicare, self-insurance and unknown. Home address was geocoded and linked to estimated median household income level by census ZIP Code Tabulation Areas [22]. Median household income was categorized as < 50,000, 50,000–99,999, 100,000–149,999, and > 150,000 dollars. Patient co-morbidities (ICD-10 diagnoses) known to affect COVID-19 severity were extracted. Comorbidities included: cancer, diabetes, hypertension, obesity, asthma, chronic obstructive pulmonary disease (COPD), and “other respiratory diseases”. Comorbidity Index (CCI) scores [23] were calculated to determine severity of comorbidities. Patients were divided into four groups: no major comorbidity (CCI = 0); mild (CCI = 1–2); moderate (CCI = 3–4); or severe (CCI ≥ 5) [23]. We identified smoking status (never, current, former smoker and unknown). Lastly, body mass index (BMI) (categorized as: underweight ≤ 18.5, 18.5 < normal weight ≤ 25, 25 < overweight ≤ 30, or obese > 30) was calculated.

### Outcomes

We describe the percentage of all patients who were tested, and who tested positive by race/ethnicity. Among COVID-19 cases, we described outcomes of hospitalization (Yes/No), admission to an ICU (Yes/No) and death (Yes/No).

### Statistical analysis

We audited the data for quality and completeness including missing data patterns; evaluated distributions to ensure that they met the assumptions of planned analyses and examined the variable distributions to detect outliers. All inferential tests were carried out at a two-tailed alpha level of 0.05. Unadjusted and adjusted Odds Ratios (ORs) were estimated for measuring effect and Chi-square tests were used for testing differences in the severity of COVID-19 and patient demographic and clinical characteristics across the Asian Americans and NH/PIs subgroups and NHW. In lieu of individual comorbidities, our final model included the CCI scores because CCI is the most commonly used comorbidity indices and includes sixteen diseases with different weights based on the strength of their association with mortality [23]. Among Asian Americans and NH/PIs and NHW COVID-19 cases, variables were assessed as potential predictors of hospitalization using multivariable logistic regression. We then conducted a subgroup analysis of hospitalized patients to assess independent predictors of ICU admission and death using multivariable logistic regression. In the multivariable logistic regression models, the median household income was entered as a continuous variable with 1 unit equals to 10,000 dollars. All analyses were performed using SAS (v9.4). This work was reviewed and approved by the Sutter Health IRB (SHIRB Study #: 2020.067EXP), granted a Waiver of Health Insurance Portability and Accountability Act Authorization and a Waiver of Informed Consent as a data-only study, and all methods were conducted in accordance with the Declaration of Helsinki.

## Results

### Distribution of race/ethnicity of patients tested for COVID-19 and with positive tests

Five hundred fifty-six thousand six hundred ninety patients were tested for SARS-CoV-2 during the study period (Table 1). NHW made up 49.6% of those tested; Hispanics, 19.6%; and non-Hispanic Blacks, 6.0%. The total Asian Americans and NH/PIs population comprised 13.8% of those tested, which was lower than the percentage of Asian patients (16.5%) in Sutter Health as of 2020. When analyzed by Asian Americans and NH/PIs subgroup, Chinese comprised 3.1%, Asian Indians 2.8%, and “other Asians” comprised 2.8% of the tested population. (Table 1).

**Table 1:** COVID-19 testing rates and results by race/ethnicity including Asian subpopulations (2/20/2020–3/21/2021; *n* = 556,690)

	**COVID-19 Testing**				**All (*N* = 556,690)**	**ColPctn (%)**
	**Negative (*N* = 486,126)**		**Positive (*N* = 70,564)**			
	**N**	**%**	**N**	**%**	**N**	
**Race**						
**Unknown**	30,267	84.2	5684	15.8	35,951	6.5
**Non-Hispanic White**	252,053	91.3	24,035	8.7	276,088	49.6
**Hispanic**	83,780	76.9	25,145	23.1	108,925	19.6
**Non-Hispanic Black**	29,628	88.5	3859	11.5	33,487	6.0
**Other Non-Hispanic Single Race Groups**	16,688	83.3	3353	16.7	20,041	3.6
**Other Mixed Race Groups**	4659	87.8	647	12.2	5306	1.0
**All Non-Hispanic AAPI**						
Asian Indian	13,803	88.8	1742	11.2	15,545	2.8
Chinese	16,288	95.8	709	4.2	16,997	3.1
Filipino	11,029	86.5	1723	13.5	12,752	2.3
Japanese	2743	95	143	5	2886	0.5
Korean	1766	95.5	83	4.5	1849	0.3
Vietnamese	2223	89.2	270	10.8	2493	0.4
Other Asian	13,699	87.5	1961	12.5	15,660	2.8
Pacific Islander	2456	79.9	616	20.1	3072	0.6
AAPI Mixed Race Group	5044	89.5	594	10.5	5638	1.0
All	69,051	89.8	7841	10.2	76,892	13.8
**All**	**486,126**	**87.3**	**70,564**	**12.7**	**556,690**	**100**

Of the entire tested population, 70,564/556,690 (12.7%) tested positive for SARS-CoV-2. Race/ethnicity-specific SARS-CoV-2 positivity rates indicate that 8.7% of the White; 23.1% of the Hispanic; 11.5% of the Black; and 10.2% of the Asian Americans and NH/PIs population tested positive. SARS-CoV-2 positivity of Asian subgroups varied from 4% in the Chinese and Korean populations, to 11.2%, 13.5%, and 12.5% for Asian Indian, Filipino, and “other Asian” populations respectively. Pacific Islanders had the greatest subgroup test positivity at 20.1%.

### Characteristics of non-Hispanic White and Asian Americans and NH/PIs populations with COVID-19 disease

Population characteristics differed between Asian Americans and NH/PIs compared to NHW with COVID-19 disease. Asian Americans and NH/PIs who tested positive were younger than NHW counterparts (Asian Americans and NH/PIs: Mean = 44.3, SD = 21.6; NHW: Mean = 49.1, SD = 21.9, *p *< 0.0001) (Table 2). Over 60% of Asian Americans and NH/PIs compared to 56.5% of NHW had commercial insurance while 14.6% of the Asian Americans and NH/PIs compared to 24.7% of the NHW population had Medicare (*p* < 0.0001). A larger proportion (14.3%) of the Asian Americans and NH/PIs population lived in a neighborhood with a median household income of $100,000 and above compared to 10.8% of NHW (*p* < 0.0001). Compared with NHW, the Asian Americans and NH/PIs population with COVID-19 had a greater prevalence of diabetes but the NHW population had a greater prevalence of cancer, hypertension, COPD, and obesity. Overall, 11.6% of NHW population had the severe major comorbidities compared to 8.3% of Asian Americans and NH/PIs (*p* < 0.0001). In terms of outcomes, the Asian Americans and NH/PIs population compared to NHW had increased proportions of hospitalization (15.1% vs. 13.8%, *p* = 0.002). Among those who were hospitalized, proportions of ICU admission and death for Asian Americans and NH/PIs vs. NHW were 25.8% vs. 25.2% (*p* = 0.681), and 22.7% vs. 24.1% (*p* = 0.332), respectively.

**Table 2:** Characteristics of AAPI compared with NHW populations with COVID-19 infection (total *n* = 31,876)

	**All Lab-confirmed Positive cases**		**Non-Hispanic White**		**All Non-Hispanic AAPI**		**All AAPI vs. NHW**	**AAPI Subgroups**																		**AAPI Subgroups**
								**1. Asian Indian**		**2. Chinese**		**3. Filipino**		**4. Japanese**		**5. Korean**		**6. Vietnamese**		**7. Other Asian**		**8. Pacific Islander**		**9. AAPI Mixed Race group**		
	**(*N* = **31,876)		**(*N* = **24,035)		**(*N* = **7841)			**(*N* = **1742)		**(*N* = **709)		**(*N* = **1723)		**(*N* = **143)		**(*N* = **83)		**(*N* = **270)		**(*N* = **1961)		**(*N* = **616)		**(*N* = **594)		
	**Mean or N**	**Std or %**	**Mean or N**	**Std or %**	**Mean or N**	**Std or %**	***P* Value**	**Mean or N**	**Std or %**	**Mean or N**	**Std or %**	**Mean or N**	**Std or %**	**Mean or N**	**Std or %**	**Mean or N**	**Std or %**	**Mean or N**	**Std or %**	**Mean or N**	**Std or %**	**Mean or N**	**Std or %**	**Mean or N**	**Std or %**	***P* Value**
**Age in Years**	47.9	21.9	49.1	21.9	44.3	21.6	< 0.0001	39.4	19.8	52.8	24.1	48.6	20.1	64.3	21.7	53.8	20.5	46.6	18.8	43.3	21.2	42.9	19.2	33.7	23	< 0.0001
**Sex**							0.046																			< 0.0001
**Female**	17,491	54.9	13,112	54.6	4379	55.8		895	51.4	394	55.6	1010	58.6	89	62.2	53	63.9	172	63.7	1081	55.1	361	58.6	324	54.5	
**Male**	14,385	45.1	10,923	45.4	3462	44.2		847	48.6	315	44.4	713	41.4	54	37.8	30	36.1	98	36.3	880	44.9	255	41.4	270	45.5	
**Age Group**							< 0.0001																			< 0.0001
** < 80**	2839	8.9	1933	8	906	11.6		262	15	64	9	122	7.1	5	3.5	4	4.8	18	6.7	200	10.2	55	8.9	176	29.6	
**18–39**	8783	27.6	6334	26.4	2449	31.2		645	37	146	20.6	434	25.2	16	11.2	17	20.5	70	25.9	721	36.8	218	35.4	182	30.6	
**40–59**	10,045	31.5	7498	31.2	2547	32.5		562	32.3	205	28.9	644	37.4	34	23.8	33	39.8	118	43.7	577	29.4	220	35.7	154	25.9	
**60–79**	7724	24.2	6272	26.1	1452	18.5		220	12.6	181	25.5	408	23.7	50	35	19	22.9	51	18.9	354	18.1	110	17.9	59	9.9	
**80 + **	2485	7.8	1998	8.3	487	6.2		53	3	113	15.9	115	6.7	38	26.6	10	12	13	4.8	109	5.6	13	2.1	23	3.9	
**Insurance**							< 0.0001																			< 0.0001
**Commercial insurance**	18,402	57.7	13,574	56.5	4828	61.6		1265	72.6	383	54	1118	64.9	76	53.1	50	60.2	157	58.1	989	50.4	339	55	451	75.9	
**Medicaid**	2511	7.9	1783	7.4	728	9.3		147	8.4	36	5.1	93	5.4	2	1.4	4	4.8	38	14.1	280	14.3	83	13.5	45	7.6	
**Medicare**	7087	22.2	5943	24.7	1144	14.6		156	9	181	25.5	301	17.5	59	41.3	22	26.5	37	13.7	260	13.3	72	11.7	56	9.4	
**Self-paying or Other/Not Reported**	3876	12.2	2735	11.4	1141	14.6		174	10	109	15.4	211	12.2	6	4.2	7	8.4	38	14.1	432	22	122	19.8	42	7.1	
**Median Household Income**							< 0.0001																			< 0.0001
**< $50,000**	6024	18.9	4721	19.6	1303	16.6		205	11.8	101	14.2	168	9.8	15	10.5	7	8.4	37	13.7	586	29.9	110	17.9	74	12.5	
**$50,000–99,999**	21,842	68.5	16,473	68.5	5369	68.5		1082	62.1	387	54.6	1420	82.4	85	59.4	52	62.7	198	73.3	1249	63.7	469	76.1	427	71.9	
**$100,000–149,999**	3396	10.7	2355	9.8	1041	13.3		419	24.1	196	27.6	120	7	38	26.6	22	26.5	31	11.5	112	5.7	32	5.2	71	12	
** > = $150,000**	318	1	236	1	82	1		26	1.5	20	2.8	4	0.2	3	2.1	1	1.2	2	0.7	8	0.4			18	3	
**Unknown**	296	0.9	250	1	46	0.6		10	0.6	5	0.7	11	0.6	2	1.4	1	1.2	2	0.7	6	0.3	5	0.8	4	0.7	
**CCI**							< 0.0001																			< 0.0001
**No comorbidity: CCI = 0**	6196	19.4	4466	18.6	1730	22.1		535	30.7	158	22.3	336	19.5	24	16.8	20	24.1	66	24.4	347	17.7	92	14.9	152	25.6	
**Mild: CCI = 1–2**	9468	29.7	7127	29.7	2341	29.9		482	27.7	208	29.3	593	34.4	43	30.1	27	32.5	74	27.4	522	26.6	193	31.3	199	33.5	
**Moderate: CCI = 3–4**	2918	9.2	2306	9.6	612	7.8		84	4.8	85	12	189	11	18	12.6	7	8.4	15	5.6	123	6.3	58	9.4	33	5.6	
**Severe: CCI > = 5**	3430	10.8	2778	11.6	652	8.3		81	4.6	82	11.6	177	10.3	32	22.4	9	10.8	25	9.3	136	6.9	73	11.9	37	6.2	
**Unknown**	9864	30.9	7358	30.6	2506	32		560	32.1	176	24.8	428	24.8	26	18.2	20	24.1	90	33.3	833	42.5	200	32.5	173	29.1	
**Smoking Status**							< 0.0001																			< 0.0001
**Current smoker**	1961	6.2	1669	6.9	292	3.7		38	2.2	16	2.3	70	4.1	10	7	3	3.6	7	2.6	76	3.9	39	6.3	33	5.6	
**Former smoker**	6440	20.2	5428	22.6	1012	12.9		110	6.3	83	11.7	339	19.7	47	32.9	16	19.3	28	10.4	192	9.8	119	19.3	78	13.1	
**Non-smoker**	20,249	63.5	14,689	61.1	5560	70.9		1440	82.7	508	71.7	1148	66.6	82	57.3	54	65.1	189	70	1310	66.8	376	61	453	76.3	
**Unknown**	3226	10.1	2249	9.4	977	12.5		154	8.8	102	14.4	166	9.6	4	2.8	10	12	46	17	383	19.5	82	13.3	30	5.1	
**Obesity**							< 0.0001																			< 0.0001
**Unknown**	12,762	40	9441	39.3	3321	42.4		616	35.4	294	41.5	641	37.2	36	25.2	30	36.1	112	41.5	1102	56.2	289	46.9	201	33.8	
**Underweight (BMI < 18.5)**	962	3	612	2.5	350	4.5		94	5.4	39	5.5	54	3.1	3	2.1	2	2.4	7	2.6	61	3.1	13	2.1	77	13	
**Normal (BMI 18.5–29)**	5233	16.4	3737	15.5	1496	19.1		408	23.4	195	27.5	309	17.9	46	32.2	22	26.5	79	29.3	286	14.6	40	6.5	111	18.7	
**Overweight (BMI 25–29.9)**	6114	19.2	4582	19.1	1532	19.5		410	23.5	138	19.5	411	23.9	34	23.8	16	19.3	52	19.3	279	14.2	90	14.6	102	17.2	
**(BMI > = 30)**	6805	21.3	5663	23.6	1142	14.6		214	12.3	43	6.1	308	17.9	24	16.8	13	15.7	20	7.4	233	11.9	184	29.9	103	17.3	
**Cancer**	1318	4.1	1160	4.8	158	2	< 0.0001	31	1.8	19	2.7	37	2.1	12	8.4	1	1.2	10	3.7	18	0.9	15	2.4	15	2.5	< 0.0001
**Hypertension**	7254	22.8	5677	23.6	1577	20.1	< 0.0001	253	14.5	153	21.6	509	29.5	57	39.9	17	20.5	51	18.9	316	16.1	131	21.3	90	15.2	< 0.0001
**Type2 diabetes**	3346	10.5	2340	9.7	1006	12.8	< 0.0001	163	9.4	74	10.4	343	19.9	30	21	7	8.4	39	14.4	202	10.3	100	16.2	48	8.1	< 0.0001
**Asthma**	2490	7.8	1903	7.9	587	7.5	0.217	121	6.9	36	5.1	189	11	17	11.9	3	3.6	18	6.7	103	5.3	47	7.6	53	8.9	< 0.0001
**COPD**	3640	11.4	2912	12.1	728	9.3	< 0.0001	149	8.6	51	7.2	227	13.2	21	14.7	6	7.2	28	10.4	132	6.7	55	8.9	59	9.9	< 0.0001
**Other chronic respiratory diseases**	12,935	40.6	9750	40.6	3185	40.6	0.933	689	39.6	282	39.8	765	44.4	66	46.2	30	36.1	103	38.1	758	38.7	237	38.5	255	42.9	0.01
**Hospitalized**	4492	14.1	3305	13.8	1187	15.1	0.002	107	6.1	157	22.1	295	17.1	38	26.6	16	19.3	47	17.4	366	18.7	102	16.6	59	9.9	< 0.0001
**ICU (out of all 4492 hospitalized patients)**	1138	25.3	832	25.2	306	25.8	0.681	22	20.6	30	19.1	83	28.1	4	10.5	2	12.5	10	21.3	109	29.8	32	31.4	14	23.7	0.025
**Death (out of all 4492 hospitalized patients)**	1068	23.8	798	24.1	270	22.7	0.332	15	14	51	32.5	68	23.1	16	42.1	3	18.8	6	12.8	82	22.4	16	15.7	13	22	0.001

There were notable variations in the demographic profiles of the different Asian subpopulations with COVID-19 disease (Table 2). Asian Indians (*n* = 1742), Filipinos (*n* = 1723) and “other Asians” (*n* = 1961) comprised the largest Asian subpopulations. Chinese (*n* = 709), Pacific Islander (*n* = 616) and mixed Asian Americans and NH/PIs race (*n* = 594) comprised the next largest Asian subpopulations. Women made up almost two-thirds of Japanese (62.2%), Korean (63.9%), and Vietnamese (63.7%) subgroups. Asian Indians were the youngest single-race population with a mean age of 39.4 (SD = 19.8) years; 58.4% of the population had mild/no or unknown comorbidities. They had the highest proportion (72.6%) of commercial insurance and the lowest proportion of hospitalization (6.1%) of all single-race Asian Americans and NH/PIs subgroups. Median household income varied greatly across Asian Americans and NH/PIs subgroups- over 30% of Chinese lived in neighborhoods with a median household income of $100,000 and above per year, compared to only 5.2% of Pacific Islander, 6.1% of “other Asian”, and 7.2% of Vietnamese. The Japanese subgroup had the oldest mean age of 64.3 years (SD = 21.7) of the entire Asian Americans and NH/PIs group, the highest percentage of Medicare (41.3%), and the highest number of patients with moderate and severe co-morbidities (35.0%). In terms of outcomes, Japanese had the highest proportions of hospitalization (26.6%) and deaths among hospitalized patients (42.1%). Pacific Islanders had a mean age of 42.9 (SD = 19.2) years and one of the lowest proportions of commercial insurance (55%) (Table 2). They also had one of the highest proportions of moderate and severe co-morbidities (21.3%). Seventeen percent of Pacific Islanders were hospitalized and had the highest proportion of ICU admission (31.4%).

### Adjusted probability of COVID-19 severe outcomes of hospitalization, ICU admission or death in the total Asian Americans and NH/PIs population and by Asian subgroups

**Fig. 1:**
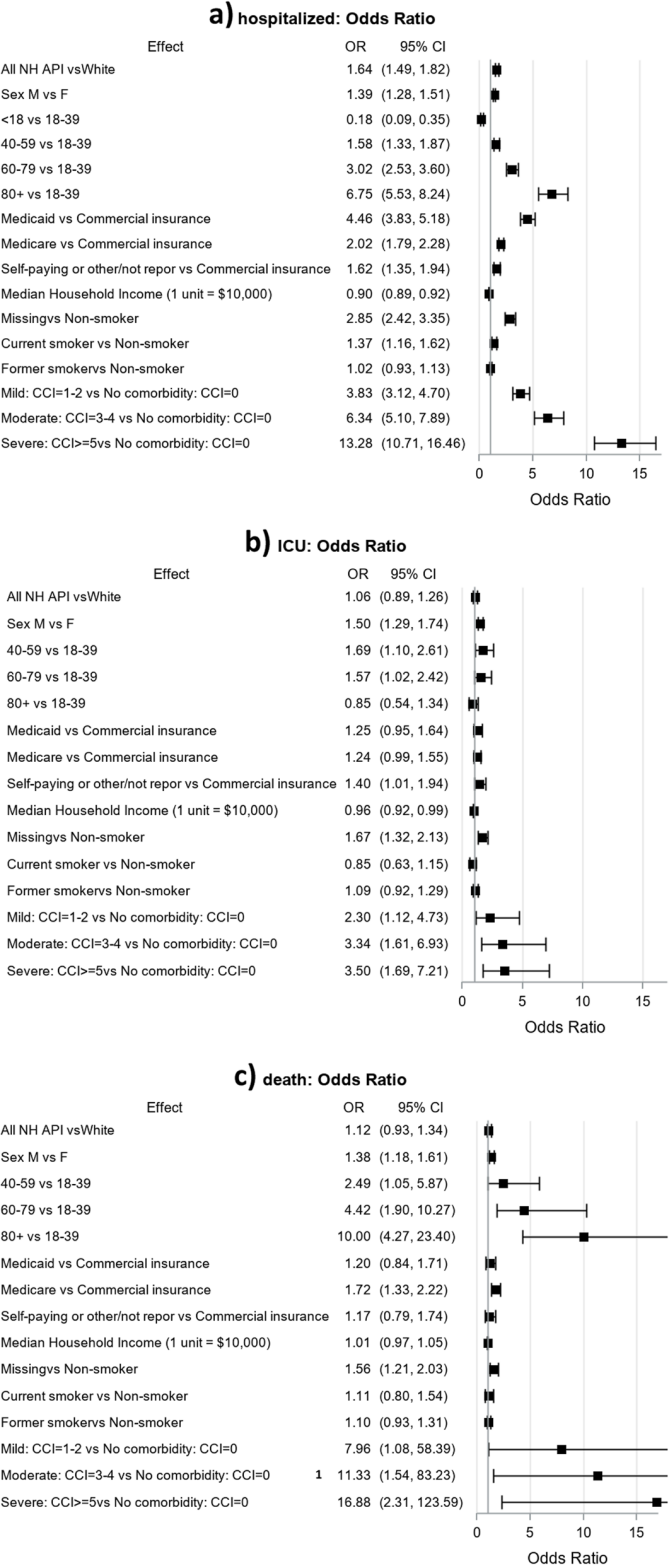
Adjusted odds ratios of COVID-19-related hospitalization, ICU admission, and death among total AAPI and NHW

In the maximally adjusted models controlling for socio-demographic information (age categories, sex, primary insurance, median household income); CCI and smoking status, the odds of Asian Americans and NH/PIs patients with COVID-19 to be hospitalized was 60% more than that of NHW (OR 1.64, 95% CI: 1.49–1.82) (Fig. 1a). Among Asian Americans and NH/PIs and NHW patients with COVID-19 disease, men were at an almost 40% higher odds of hospitalization than women. Adjusted odds of hospitalization increased with age. The odds of hospitalization for patients with Medicaid was more than 4 times that of patients with commercial insurance, two times that of patients with Medicare and more than 1.5 times that of those with no insurance; likelihood of hospitalization of patients with Medicaid was approximately double that of those with commercial insurance. For each $10,000 increase in median household income, there was a 10% decrease in the odds of hospitalization. Patients with unknown smoking status had almost three times and current smokers had almost one and a half times odds of hospitalization compared to non-smokers. Compared to the patients with no major comorbidities, those with mild, moderate, and severe chronic conditions had increasingly greater odds of hospitalization. There were no significant differences in ICU admission or death between the total population of Asian Americans and NH/PIs and NHW (Fig. 1b,c).

**Fig. 2:**
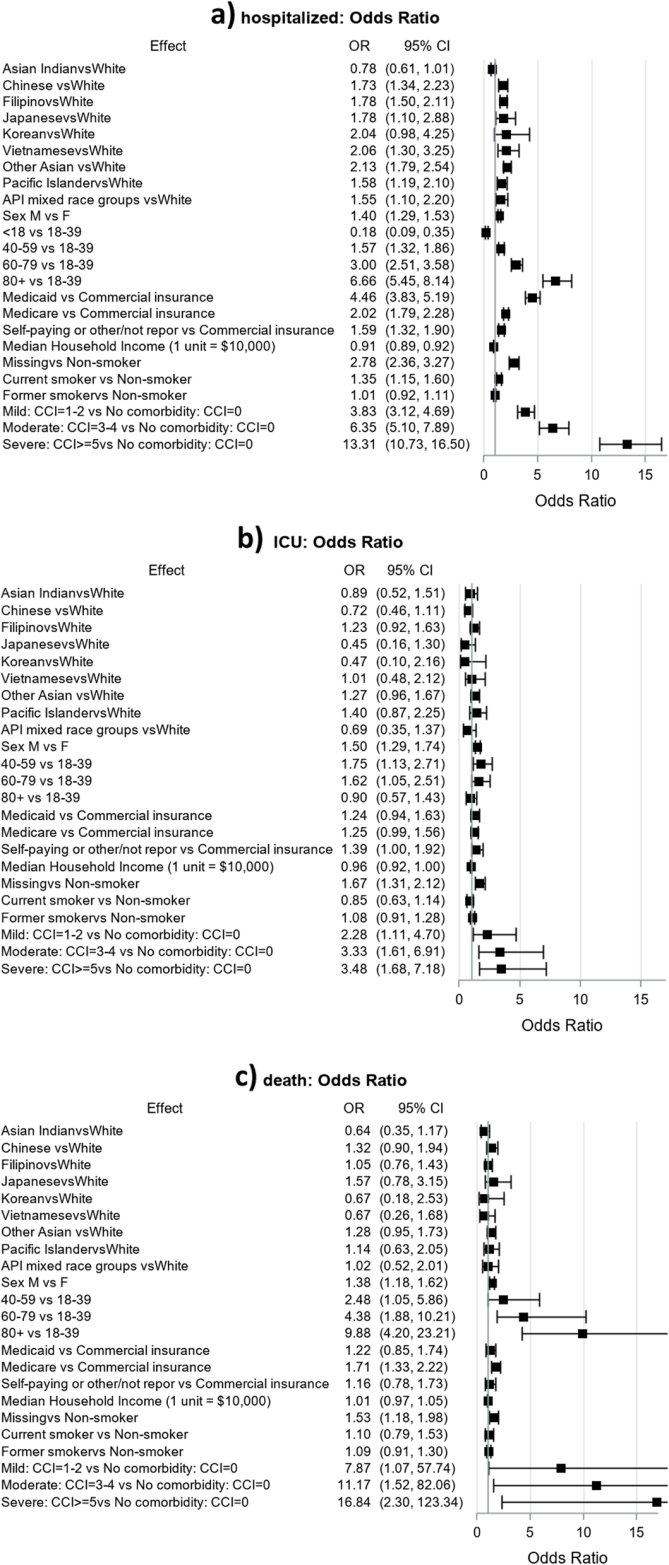
Adjusted odds ratios of COVID-19-related hospitalization, ICU admission, and death by Asian sub-populations

When analyzing the adjusted association of hospitalization by specific Asian subgroups compared to NHW, Asian subgroups showed notable variation in odds of hospitalization (Fig. 2a). Vietnamese (OR = 2.06, 95% CI = 1.30–3.25), “Other Asian” (OR = 2.13, 95% CI = 1.79–2.54), Filipino (OR = 1.78, 95% CI = 1.34–2.23), Japanese (OR = 1.78, 95% CI = 1.10–2.88), and Chinese (OR = 1.73, 95% CI = 1.34–2.23) subgroups had almost double the odds of hospitalization compared to NHW. Pacific Islander (OR = 1.58, 95% CI = 1.19–2.10) and Asian Americans and NH/PIs mixed race subgroups (OR = 1.55, 95% CI = 1.10–2.20) had more than one and a half times odds of hospitalization compared to NHW. There was no statistical difference when comparing Koreans and NHW (OR = 2.04, 95% CI = 0.98–4.25). The adjusted odds of hospitalization was lower among Asian Indians (OR = 0.78, 95% CI = 0.61–1.01) compared to NHW but the difference was not statistically significant. The contribution of socio-demographic and clinical characteristics to hospitalization was similar to the analysis using the entire Asian Americans and NH/PIs population compared to NHW (Fig. 1a). Adjusted odds of ICU admission or death among hospitalized patients by different Asian subgroups varied but were not statistically significant (Fig. 2b, c).

## Discussion

We uniquely demonstrate large variations in COVID-19 testing proportions, test positivity, and disease severity in the Asian Americans and NH/PIs population in Northern and Central California. Our disaggregated Asian subgroup data reveal previously obscured outcomes.

The total percentage of Asian Americans and NH/PIs tested at our health care sites was lower than their share of the area and health system population prevalence. This documented lack of testing suggests an undercounting of cases, and therefore an underestimate of the COVID-19 disease burden in these populations. Generally, COVID-19 testing rates by race and ethnicity are not well understood due to incomplete data collection. Reports from California regarding testing early during the pandemic and even more recently as of March 2022, were missing a substantial amount, as much as 30–40%, of race/ethnicity information [24]. Decreased testing could be due to different mechanisms. The lack of testing sites in underserved neighborhoods, difficulty locating and navigating to a testing site due to insurance type, language abilities; increased fear to travel due to fear of contracting COVID -19 and fear of anti-Asian violence have all been posited as causes for lack of testing [25–28].

The percent of test positivity was overall increased for the total Asian Americans and NH/PIs population compared to NHW but differed between Asian subgroups. Test positivity or COVID-19 infection could be affected by differential exposure to SARS-CoV-2 virus; as well as inability to protect against infection if exposed to the virus. Asian subgroups, such as Filipinos, Vietnamese, and Pacific Islanders are known to have a larger share of their population as essential workers in health care, supermarkets, restaurants, and other occupations with potentially increased exposure to the infected and decreased ability to work from home [29–31]. In the early phases of the pandemic, lack of adequate PPE was documented for many essential workers such as food workers, mass transit staff, and public safety employees.

Once infected, Asian populations who have lower household incomes may not have been able to take time off from work, or may live in crowded conditions and might not have the ability to isolate, increasing the chance of transmitting and others contracting COVID-19 disease [32]. Asian Americans and NH/PIs are more likely to live in multigenerational households compared to NHW overall, which could increase risk of COVID-19 exposure of individuals at higher risk of serious disease within the same household [33–36]. Differences among Asian subgroups in educational attainment and occupation may contribute to differences in risk of COVID-19 exposure [7, 37]. Asian Indian, Korean, and Japanese subgroups have the highest educational attainment rates among Asian Americans and NH/PIs, while, Pacific Islanders have one of the lowest educational attainment rates [38]. During the pandemic, some individuals who worked high-income jobs were more likely to be able to telework, decreasing risk of COVID-19 exposure. Although the Chinese, Japanese and Korean subgroups had a lower test positivity rate than NHW, all other Asian Americans and NH/PIs subgroups had a higher test positivity rate compared to NHW, demonstrating that subsuming all subgroups under the umbrella term of "AAPI" renders these differences in test positivity invisible.

The Asian Americans and NH/PIs population as a whole was more likely to be hospitalized compared to NHW. Furthermore, proportions of hospitalized Asian subgroups varied greatly. The likelihood of hospitalization varied from twice as likely as NHW for Chinese, Vietnamese, Filipino, Japanese, and “other Asian” to no significant difference compared to NHW for Asian Indians and Koreans. While we adjusted for the socio-demographic and clinical factors, other mechanisms to develop serious disease requiring hospitalization include delayed testing and diagnosis of COVID-19 as well as delayed access to health care until disease severity required hospitalization [39, 40]. Immigration status, length of time in the country, and primary language spoken can all affect health care access. Newly arrived immigrants or undocumented immigrants may not be eligible for insurance or fear seeking health care due to anti-Asian xenophobia [41]. Furthermore, low-income immigrants may also demonstrate hesitation toward preventative care-seeking until care is urgently necessary (prompting hospitalization ER visits, etc.) due to cost or insurance barriers, which may also lead to worsened health outcomes during the pandemic [42]. Telehealth, a touted technological solution to health care access, might not be as accessible for limited English proficient individuals and individuals with limited technology literacy. In addition, uninsured or low socioeconomic status individuals may not have access to electronic devices or reliable internet connectivity required for a quality telehealth visit [43].

Our work also demonstrates unique profiles among Asian subgroups in the context of COVID-19. We are aware of only one other analysis by Marcello et al. which analyzed outcomes of three Asian subgroups: Chinese, South Asian, and “other Asians” in the New York City public hospital system during the first three months of the pandemic [6]. Of note, this study identified the subgroup population by utilizing EHR race/ethnicity and ancestry data. They found that South Asians had the highest proportions of COVID-19 disease and hospitalization among Asians in their study population. The differences in proportions of South Asian vs. Asian Indian hospitalization between our studies is most probably secondary to the Marcello study definition of South Asian encompassing many subgroups (Afghani, Bangladeshi, Indian, Nepalese, Pakistani, Sri Lankan). This population thus was heterogeneous population including a larger proportion of essential workers and seniors among the South Asian community in New York City compared to our study’s more homogeneous, younger Asian Indian community in Northern and Central California.

We demonstrate uniquely with the COVID-19 pandemic, differential testing proportions and severe outcomes due to the fact that Asian subgroup populations have different demographic, socio-economic (including predominant immigration status and occupations), and health profiles [7, 29, 44]. Aggregated Asian data perpetuates structural perpetuation of racist stereotypes and notions of the “model minority myth” and “healthy immigrant effect” that adversely affect Asian Americans and NH/PIs health generally and during the pandemic [18]. The generalization of good health status among Asian Americans and NH/PIs oversimplifies and obscures worse health outcomes unique to different Asian subgroups. It bears mention that while the rise in anti-Asian violence has impacted the entire Asian Americans and NH/PIs community, the exacerbation of racism and xenophobia has especially impacted Chinese communities due to the initial cases of COVID-19 identified in Wuhan, China [45]. The increased fear of anti-Asian violence not only could have deterred many Asian Americans, and specifically members of the Chinese community, from seeking testing or health care, but possibly exacerbated illness, medical and mental health conditions, leading to increased mortality [41].

### Limitations and strengths

This study is an analysis of a single health system in Northern and Central California. The Asian Americans and NH/PIs population studied may not be generalizable to other populations. However, the health system encompasses the largest concentration of Asian Americans and NH/PIs in the continental US. To the best of our knowledge, this is the only large health system that has been committed to systematically disaggregating Asian Americans and NH/PIs data into the six major Asian subgroups, “other Asian”, Pacific Islander, and mixed race subgroups, providing a unique source of timely clinical data.

The San Francisco Bay Area public health systems were among the first to take committed, ongoing measures to control the pandemic in March 2020. Our COVID-19 outcomes might be much better than other areas of the US and consequently, our analyses of subgroup severe outcomes could be limited by sample size to demonstrate statistical significance, for the outcomes of ICU admission and death.

During the beginning of the pandemic, COVID-19 testing was limited and primarily conducted in individuals with symptoms and with a history of travelling to China [46]. Patients could have been tested not at all, or in venues such as trusted cultural community centers outside of the Sutter Health system [26, 47]. However, while the Sutter Health testing data might be limited, our data is a true reflection of the extent to which the patients with COVID-19 had outcomes of hospitalization, ICU admission and death. Careful analysis and interpretation of these data are the beginnings of insight in understanding the effect of the pandemic on Asian Americans and NH/PIs subgroups.

## Conclusions

We reveal for the first time, variations in COVID-19 testing, positivity, and outcomes among nine Asian Americans and NH/PIs subgroups in a large California health care system. Important nuances of the cadence of testing, positivity and disease severity are evident when looking at disaggregated Asian Americans and NH/PIs data. Our findings highlight the unique health profiles and needs of Asian Americans and NH/PIs subgroups. As the pandemic continues, it is vital to ensure that lessons learned regarding inequities in access to testing and care are not forgotten, but rather that new challenges such as access to vaccination, early and prophylactic treatment for high-risk populations including all vulnerable populations, such as Asian Americans and NH/PIs subgroups, are prioritized.

## Supplementary Information

**Additional file 1.**

**Additional file 2.**

## Data Availability

The datasets generated and/or analyzed during the current study are not publicly available due to their containing patient health information that could compromise the privacy of research participants but are available from the corresponding author on reasonable request.

## Abbreviations

Asian Americans and NH/PIs: Asian Americans, Native Hawaiians, and Pacific Islanders
NHW: Non-Hispanic Whites
EHR: Electronic health records
CURVE: COVID-19 Universal Registry for Vital Evaluations
OR: Odds Ratio
US: United States
ICU: Intensive care unit
RT-PCR: Reverse transcription polymerase chain reaction
COPD: Chronic obstructive pulmonary disease
CCI: Comorbidity Index
BMI: Body mass index
