# Supplementary material for: Disparities in COVID-19 testing and outcomes among Asian American and Pacific Islanders: an observational study in a large health care system
Source: BMC Public Health. 2023 Feb 6;23:251. doi: 10.1186/s12889-023-15089-w (PMC9900558; doi:10.1186/s12889-023-15089-w)
Supplement: Supplementary file 2 — Additional file 2. [file 12889_2023_15089_MOESM2_ESM.docx]

| **Effect** | | **Hospitalized** | | **ICU** | | **Death** | |
| --- | --- | --- | --- | --- | --- | --- | --- |
|  |  | **Unadjusted OR** | **Unadjusted 95% CI** | **Unadjusted OR** | **Unadjusted 95% CI** | **Unadjusted OR** | **Unadjusted 95% CI** |
| Race/ethnicity | Asian Indian vs. NH White | 0.41 | 0.34-0.5 | 0.36 | 0.23-0.55 | 0.25 | 0.16-0.39 |
|  | Chinese vs. NH White | 1.78 | 1.49-2.14 | 1.23 | 0.85-1.79 | 2.13 | 1.63-2.79 |
|  | Filipino vs. NH White | 1.30 | 1.14-1.48 | 1.41 | 1.12-1.78 | 1.07 | 0.85-1.36 |
|  | Japanese vs. NH White | 2.27 | 1.56-3.3 | 0.80 | 0.3-2.17 | 4.34 | 2.77-6.82 |
|  | Korean vs. NH White | 1.50 | 0.87-2.59 | 0.69 | 0.17-2.81 | 1.15 | 0.42-3.14 |
|  | Vietnamese vs. NH White | 1.32 | 0.96-1.81 | 1.07 | 0.57-2.02 | 0.78 | 0.4-1.52 |
|  | Other Asian vs. NH White | 1.44 | 1.28-1.62 | 1.64 | 1.34-2.02 | 1.12 | 0.9-1.39 |
|  | Pacific Islander vs. NH White | 1.25 | 1-1.54 | 1.53 | 1.06-2.2 | 0.72 | 0.45-1.14 |
|  | API mixed race vs. NH White | 0.69 | 0.53-0.91 | 0.67 | 0.39-1.15 | 0.63 | 0.38-1.03 |
| Sex | M vs. F | 1.31 | 1.23-1.39 | 1.86 | 1.65-2.1 | 1.57 | 1.41-1.76 |
| Age Group | <18 vs.18-39 | 0.25 | 0.18-0.36 | - | - | - | - |
|  | 40-59 vs.18-39 | 1.97 | 1.74-2.23 | 4.36 | 3.2-5.94 | 7.70 | 4.12-14.37 |
|  | 60-79 vs.18-39 | 7.15 | 6.38-8.01 | 14.58 | 10.87-19.55 | 59.47 | 32.71-108.11 |
|  | 80+ vs.18-39 | 23.32 | 20.51-26.51 | 20.56 | 15.09-28.01 | 307.77 | 169.28-559.56 |
| Insurance | Medicaid vs. Commercial | 6.39 | 5.72-7.13 | 6.58 | 5.34-8.1 | 4.59 | 3.48-6.05 |
|  | Medicare vs. Commercial | 10.17 | 9.37-11.03 | 9.39 | 8-11.03 | 22.94 | 19.14-27.5 |
|  | Self-paying/other/not reported vs. Commercial | 1.85 | 1.63-2.1 | 2.42 | 1.9-3.08 | 2.98 | 2.26-3.91 |
| Income | Median household income (1 unit=$10,000) | 0.88 | 0.86-0.89 | 0.88 | 0.86-0.89 | 0.88 | 0.86-0.89 |
| Smoking | Missing vs Non-smoker | 1.69 | 1.53-1.87 | 2.23 | 1.86-2.66 | 1.71 | 1.43-2.06 |
|  | Current smoker vs Non-smoker | 1.76 | 1.55-1.99 | 1.81 | 1.43-2.29 | 1.29 | 1-1.66 |
|  | Former smoker vs Non-smoker | 2.39 | 2.22-2.58 | 2.57 | 2.24-2.95 | 3.27 | 2.9-3.69 |
| Severity of Major Comorbidities | Mild: CCI=1-2 vs No comorbidities | 8.29 | 6.81-10.08 | 21.79 | 11.22-42.33 | 21.45 | 10.61-43.36 |
|  | Moderate: CCI=3-4 vs No comorbidities | 22.85 | 18.65-27.99 | 64.96 | 33.36-126.47 | 80.96 | 40.06-163.63 |
|  | Severe: CCI>=5 vs No comorbidities | 57.38 | 47.05-69.98 | 118.92 | 61.44-230.19 | 219.72 | 109.4-441.29 |

**Supplemental Table 2.** **Unadjusted odds ratios of COVID-19-related hospitalization, ICU admission, and death by Asian sub-populations**
